# Safety and Efficacy of Low-Dose Eptifibatide for Tandem Occlusions in Acute Ischemic Stroke

**DOI:** 10.3390/neurolint16010017

**Published:** 2024-02-09

**Authors:** Paweł Latacz, Tadeusz Popiela, Paweł Brzegowy, Bartłomiej Lasocha, Krzysztof Kwiecień, Marian Simka

**Affiliations:** 1 Department of Vascular Surgery and Angiology, Brothers of Mercy St. John of God Hospital, 31-061 Krakow, Poland; pawlat@me.com (P.L.); krzyskwiecien@yahoo.com (K.K.); 2 Chair of Radiology, Jagiellonian University Medical College, 31-008 Krakow, Poland; msjpopie@cyf-kr.edu.pl (T.P.); pawelbrzegowy007@gmail.com (P.B.); 3 Diagnostic Imaging Unit, University Hospital, 31-501 Krakow, Poland; blasocha@su.krakow.pl; 4 Institute of Medical Sciences, University of Opole, 45-040 Opole, Poland

**Keywords:** ischemic stroke, tandem lesion, epifibatide, stent

## Abstract

Objectives: The optimal treatment strategy for ischemic stroke in patients presenting with tandem occlusions of the internal carotid artery remains controversial. Several studies have demonstrated better clinical outcomes after eptifibatide, which is a short half-life antiplatelet agent. This retrospective analysis focused on the safety and efficacy of low-dose eptifibatide administration in stroke patients with tandem lesions. Methods: We evaluated the results of endovascular treatment in 148 stroke patients with tandem lesions. Patients in whom balloon angioplasty alone resulted in satisfactory cerebral flow did not receive eptifibatide (33 patients); others received this drug together with stent implantation (115 patients). Eptifibatide was given as an intravenous bolus of 180 μg/kg and then in a modified low dose of 1 μg/kg/min for 24 hours. Results: There were no statistically significant differences between both groups regarding 30-day mortality, frequency of thrombotic events, or hemorrhagic complications. An analysis of clinical status at 30-day follow-up revealed that the administration of eptifibatide was associated with a statistically significant better outcome: a higher rate of either no neurological symptoms or only mild symptoms (4 NIHSS points maximally). Conclusions: The administration of eptifibatide in stroke patients presenting with tandem lesions is relatively safe. Moreover, treatment with this drug can improve clinical outcomes in these challenging patients.

## 1. Introduction

The optimal treatment strategy for acute ischemic stroke in patients presenting with so-called tandem lesions—i.e., comprising both occlusion or high-grade stenosis of the extracranial segment of the internal carotid artery (ICA) coexisting with occlusion of the intracranial cerebral arteries (intracranial segments of the ICA, the middle, or the anterior cerebral artery)—still remains controversial [1,2,3]. Data that come from clinical trials are scarce, since such patients typically represent only 10–20% of the cohorts studied. Quite often, patients presenting with tandem lesions are excluded from randomized controlled trials [4,5,6,7]. However, it is known that intravenous trombolysis in these patients is able to recanalyze occlusions in only about 5% of the cases, while the risk of hemorrhagic complications is high. Moreover, good clinical outcomes after standard intravenous trombolysis in stroke patients with tandem lesions are at the level of 17% with a mortality rate at the level of 50% [8,9]. On the other hand, data coming from observational studies suggest that reperfusion rates and clinical outcomes are better if extracranial lesions in these patients are managed with angioplasty and stenting [9,10,11,12].

Antiplatelet therapy is another controversial issue associated with the treatment of strokes in patients presenting with tandem lesions [11,12,13,14,15]. Recommended pharmacological treatments accompanying elective carotid stent placement include dual-antiplatelet therapy and intraprocedural anticoagulation. Yet, such a strategy is controversial in the setting of acute stroke because of the risk of intracranial hemorrhage, which is especially high if heparin is used. Nonetheless, current guidelines also recommend antiplatelet agents in these patients, particularly intravenous aspirin, although good-quality data regarding safety profile and clinical efficacy are needed [16].

This risk of intracranial hemorrhage in stroke patients particularly concerns tandem occlusions. An increased risk of intracranial bleeding is also seen when antiplatelet agents are used in these patients. Therefore, usually, these pharmacological agents are not used at the beginning of treatment. Moreover, bleeding associated with long half-life antiplatelet agents, such as aspirin or clopidogrel, cannot be easily controlled and is almost always associated with neurologic deterioration and poor clinical outcomes [10,11,14,15,17,18,19].

This problem could be overcome if a short half-life drug was used. Theoretically, the early administration of such an antiplatelet agent in patients managed with angioplasty and stenting should decrease the risk of intraprocedural cerebral embolic events as well as the risk of stent thrombosis. For this purpose, GP IIB/IIIA receptor antagonists have been used with the most common agent being eptifibatide.

Epifibatide is cleared by the kidneys, and its pharmacokinetic plasma half-life is about 4 h. Because of its short half-life, in patients without renal insufficiency, a cessation of epifibatide infusion relatively quickly restores normal hemostatic platelet function [20]. Its biological half-life is estimated to be at the level of 2.5 h, while the “real-life” stop of a prolonged bleeding, demonstrated in many clinical trials and in every-day hospital practice, is within 30 min. It should be noted that eptifibatide is contraindicated in patients undergoing hemodialysis. It is also contraindicated in patients with thrombocytopenia with a platelet count of less than 100,000/μL. This drug is primarily used in patients receiving coronary angioplasty and presenting with unstable angina or non-ST elevation myocardial infarction. Another current clinical application of epifibatide is the management of acute ischemic stroke especially in patients presenting with tandem lesions. In these patients, there is a high risk of thrombotic reocclusion, while the risk of hemorrhagic transformation of ischemic necrosis is also high. Thus, there is a need for a short half-life and a reversible antiplatelet drug.

Yet, data regarding the safety and efficacy of early administration of eptifibatide in this particular group of stroke patients are scarce and inconclusive [18,19,21,22,23]. Moreover, according to the producer, this pharmacological agent is contraindicated in stroke patients; thus, all published reports on eptifibatide in stroke patients concern its off-label administration. A meta-analysis by Liu et al. revealed that the treatment of stroke patients with eptifibatide neither had an effect on favorable neurological outcomes nor did it increase mortality [24]. On the other hand, several studies have demonstrated that treatment with eptifibatide in stroke patients can be relatively safe and can improve stroke outcomes. It is possible that these dissimilar observations of this agent resulted from its different dosing. Accompanying coronary stent implantation, eptifibatide is typically administered as an intravenous infusion at a rate of 2 μg/kg/min. It has already been suggested that perhaps such dosing in stroke patients is too high, and indeed, in several trials, it has been lowered to 0.75 μg/kg/min or 1 μg/kg/min.

The aim of this single-center retrospective analysis was to assess the safety and efficacy of the administration of the modified low dosage of eptifibatide (Integrelin, GlaxoSmithKline, Brentford, UK) accompanying endovascular angioplasty and stenting of the ICA in patients presenting with tandem lesions in the acute phase of ischemic stroke. This drug was administered in the standard initial intravenous bolus of 180 μg/kg, but instead of the usually recommended infusion at the rate of 2 μg/kg/min, a dose of 1 μg/kg/min was used. This modification was primarily aimed at reducing potential hemorrhagic complications.

## 2. Materials and Methods

**Figure 1:**
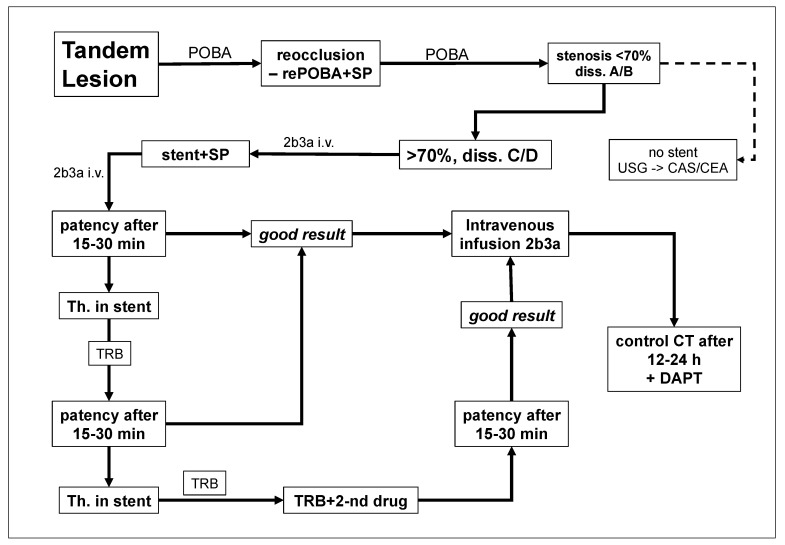
Algorithm of the management of stroke patients with tandem lesions. POBA—balloon angioplasty; SP—protection system; diss. (A/B or C/D)—dissection type A–D); 2b3a i.v.—eptifibatide intravenous infusion; patency after 15–30 min.—control angiography after 15–30 min in cathlab; Th. in stent—thrombosis in stent; TRB—thrombectomy; TRB+2-nd drug—thrombectomy + second antiplatelet drug; DAPT—dual antiplatelet treatment; control CT—CT after 12–24 h.

This study is a post hoc analysis, which focused on the safety of the early administration of eptifibatide in stroke patients with tandem lesions. We retrospectively evaluated the results of the endovascular treatment in 148 consecutive patients (114 men and 34 women) presenting with acute ischemic stroke due to tandem lesions, who were managed from 2016 to 2023. All of them were treated following our standard hospital protocol for the management of ischemic stroke caused by tandem lesions (Figure 1). This survey includes both patients with strokes of atherosclerotic etiology and those presenting with stroke due to artery dissection.

Patients were aged 23–98 years (mean: 65 ± 12.8). Overall, 111 patients (75%) presented with total occlusion of the ICA, while the remaining 37 patients (25%) had 80–99% stenosis of this artery. All of them had also occlusions in the intracranial arteries supplying the brain—details are given in (Table 1).

The primary endpoint of this study was the proportion of patients who had 30-day mortality of any cause, any intracranial bleeding or any periprocedural complications comprising target artery dissection, perforation and/or new embolic cerebral infarction. Symptomatic intracranial hemorrhage, according to the European Cooperative Acute Stroke Study II, was defined as any intraparenchymal, subarachnoid or intraventricular bleeding, which was associated with clinical worsening of at least 4 points in the National Institutes of Health Stroke Scale (NIHSS). Stent thrombosis and/or hemorrhagic complications were diagnosed either intraprocedurally, or during routine CT or MR imaging, which was performed 24 h after the procedure, or during diagnostics that was started immediately in a case of clinical worsening or recurrence of neurologic symptoms. For the latter purpose, Doppler sonography together with CT or MR angiography were utilized. The secondary endpoint of this study was neurological status measured with the NIHSS at 30-day follow-up.

For the purpose of this survey, all patients were divided into two groups: (i) receiving a short half-life GP IIB/IIIA receptor antagonist (eptifibatide) intra- and postprocedurally, and (ii) patients in whom antiplatelet treatment was postponed until the second postprocedural day, when—after exclusion of intracranial bleeding—all patients were allowed to receive standard antiplatelet therapy if such a therapy was clinically justified. No patients received heparin during endovascular procedure.

Decision upon the administration of eptifibatide was primarily associated with early stent implantation. If endovascular angioplasty of extracranial occlusion/stenosis of the ICA, using a long inflation of the balloon, resulted in satisfactory dilatation of the artery and good inflow (there should be less than 70% residual stenosis of the extracranial lesion after primary angioplasty (Figure 1) and good inflow to the cerebral arteries), the decision upon stent implantation was postponed until 2–7 days postprocedure. Otherwise, if angioplasty failed or there was an immediate reocclusion, stent was implanted and eptifibatide was administered. This agent was given in an initial intravenous bolus of 180 μg/kg (total dose of such a bolus in our patients: 11–16 mg) and then as intravenous infusion 1 μg/kg/min for 24 h. Eptifibatide was also administered in the same doses if there were the above-described indications for stent implantation, yet the stent was not implanted due to technical problems; in this case series, there were two such patients. Given these indications, patients receiving eptifibatide presented with an unfavorable morphology of tandem lesions, potentially with a high risk of thrombotic reocclusion, which justified antiplatelet treatment. Of note, the reduced dose of eptifibatide—1 μg/kg/min instead of standard 2 μg/kg/min—was primarily aimed at the reduction in hemorrhagic complications.

Out of the total of 148 patients assessed in this survey, 115 individuals (78%) received eptifibatide. In this group, 111 patients had stent implantation, including 7 patients in whom two stents were implanted. The remaining 33 patients (22%) did not receive early antiplatelet treatment and had no stents implanted.

The clinical and radiologic characteristics of strokes in both groups (i.e., receiving or not receiving early antiplatelet therapy) were similar. Median scores of the NIHSS at the baseline were 15 and 16, respectively, and this difference was statistically insignificant (see Table 1). The average ASPECT score (the Alberta stroke program early CT score) in patients receiving eptifibatide was 7.7, while in those without antiplatelet treatment, it was 7.3. Similarly, postprocedural recanalization rates of the middle cerebral artery in both groups were comparable. The Thrombolysis in Cerebral Infarction (TICI) score in patients receiving eptifibatide was TICI2b in 20% of them, TICI2c in 8%, and TICI3 in 72%. In patients without antiplatelet treatment, these scores were TICI2b in 27% of the patients, TICI2c in 12%, and TICI3 in 61%. Mean volumes of the necrosis and penumbra in patients receiving eptifibatide were 16.2 mL and 114.3 mL, respectively, while in those without antiplatelet treatment, the mean volumes were 28.6 mL and 135.8 mL, respectively. The difference between the groups regarding necrosis volume was statistically significant (*p* = 0.01; assessed using the two-sample *t*-test), while the difference in terms of penumbra volume was statistically insignificant. The preprocedural clinical characteristics of the patients are given in Table 1.

The algorithm of patients’ management is presented in Figure 1. Extracranial occlusions or stenoses of the ICA were managed with balloon angioplasty with or without stent implantation. In addition, all patients received standard intravenous thrombolysis with alteplase. Decision upon stent implantation, as well as upon the administration of antiplatelet agent was left to the discretion of doctor performing the endovascular procedure. For all patients, the team tried to perform thrombectomy of the intracranial occlusion first and to address extracranial lesion thereafter [25]. If introduction of the reperfusion catheter into the distal segments of the ICA was not possible, the proximal critical lesion was managed firstly either with angioplasty alone or with angioplasty and stenting.

Protection systems were used in 44 patients. The proximal protection devices used comprised the Mo.Ma Ultra 8F (Medtronic, Minneapolis, MN, USA) and the Cello (Covidien, ev3 Endovascular, Inc., Plymouth, MN, USA); the SpiderFX™ Embolic Protection Device (Medtronic, Minneapolis, MN, USA) was used as a distal protection system. Decision upon distal or proximal protection depended on the morphology of occluding lesions as well as on the angioarchitecture of the carotid arteries. Stents were implanted if balloon angioplasty alone failed to reopen the extracranial occlusion or if the flow improvement in this segment lasted only a few minutes and control intraprocedural angiography revealed re-occlusion of the artery. We used several types of stents: the Carotid WallstentTM (Boston Scientific, Natick, MA, USA), the RoadSaver™ stent (Terumo, Tokyo, Japan), and the XACT stent (Abbott Vascular, Abbott Park, IL, USA). The type, length and diameter of the stents were tailored to the angioarchitecture of the target lesion.

In order to reveal potential reocclusion and/or bleeding control, intraprocedural angiography was performed in each patient 5, 10 and 15 min after the stent implantation. The angiographic success of the procedure was evaluated using the modified Treatment in Cerebral Infarction score (mTICI) with the assessment of the previously occluded part of cerebral circulation at the end of endovascular repair.

In all patients, in order to assess the efficacy of the treatment, patency of the stent and/or target artery, and to reveal potential early intracranial bleeding, CT or MR of the head was performed 24 h after the procedure. Then, if there were no hemorrhagic complications, patients were recommended to take dual antiplatelet therapy (aspirin + clopidogrel or ticagrelor) for 1–3 months.

### Statistical Analysis

In order to compare the preprocedural clinical characteristics of the patients, as well as the rates of severe complications in both groups, such as bleeding, thrombosis or death, Fisher’s exact test was used. To compare parametric pre-interventional neurologic status (NIHSS scores, ASPECT score, TICI score, necrosis volume) and the duration of hospital stay, we used the two-sample t-test; in the latter case, patients who died were excluded. To compare neurologic status at 30-day follow-up, we categorized the patients into six ordinal groups: without neurologic symptoms (NIHSS score: 0), with mild stroke symptoms (NIHSS score: 1–4), moderate stroke symptoms (NIHSS score: 5–14), severe stroke symptoms (NIHSS score: 15–24), very severe stroke symptoms (NIHSS score: 25 and more), and those who died for any reason. Significance of the differences regarding neurologic status at the 30-day follow-up was calculated using the Mann–Whitney U test. In order to assess the correlation between the pre-procedural necrosis volume and the NIHSS score at the 30-day follow-up, Pearson’s r coefficient was calculated. The significance of the *p* values of all statistical tests used was set at *p* < 0.05. Statistical analysis was performed using the PAST data analysis package (version 3.0; University of Oslo, Oslo, Norway).

## 3. Results

Clinical assessment at 30-day follow-up was possible in all patients in this series except for those who died. There were 12 (8.1%) fatalities: 9 (7.8%) in the group of patients receiving eptifibatide and 3 death (9.1%) in the group of patients who were not managed with this drug. This difference was statistically insignificant. Of note, the frequencies of thrombotic and hemorrhagic complications were higher in patients not receiving eptifibatide, yet these differences were not statistically significant. The duration of hospital stay was similar in both groups of patients. Baseline neurological status measured with the NIHSS was almost the same in both groups (15 vs. 16 points). Yet, at the 30-day follow-up, the clinical status of the patients who received eptifibatide was better (median NIHSS: 3 vs. 7 points). Details are given in Table 2.

**Figure 2:**
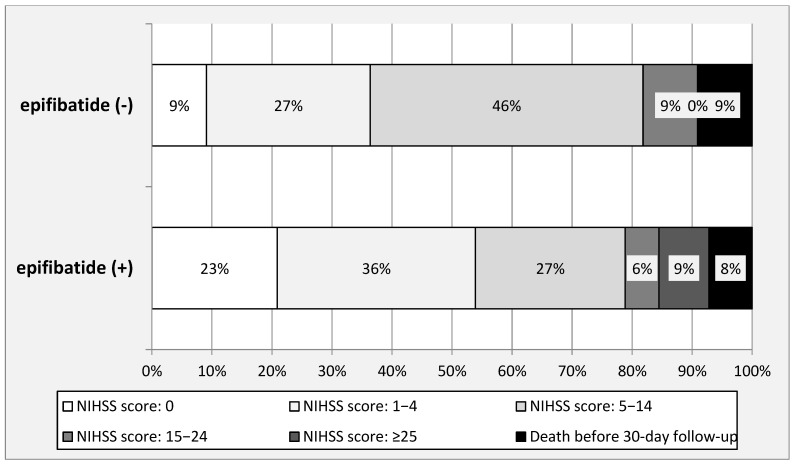
Neurological status measured by National Institutes of Health Stroke Scale at 30-day follow-up; upper graph—patients not receiving eptifibatide, lower graph—patients receiving eptifibatide. Differences regarding clinical outcome were statistically significant (*p* = 0.04; Mann–Whitney U-test) in favor of patients managed with eptifibatide.

Detailed analysis of neurological status at the 30-day follow-up has revealed that the early administration of this short half-life antiplatelet agent in a modified lower dose was associated with a higher rate of either no neurological symptoms or symptoms of a mild stroke (maximally 4 NIHSS points). This beneficial effect of the modified early administration of eptifibatide was statistically significant, while the rates of severe adverse events, including fatalities, were not significantly different from those in the group of patients not receiving such a medication. Details of this analysis are given in Table 3 and also shown in Figure 2. Since there was a statistically significant difference between the groups regarding baseline necrosis volume, we have checked whether this parameter was correlated with the clinical outcome. Still, Pearson’s r coefficient was 0.21, revealing a weak correlation only.

## 4. Discussion

In this retrospective analysis, we have found that the administration of eptifibatide in stroke patients presenting with tandem lesions was relatively safe. The rates of thromboses of the target artery, as well as the rates of intracranial hemorrhages, were not statistically different from those in the group of patients not receiving this antiplatelet agent. The mortality rate was not significantly higher after administration of eptifibatide, either. The frequencies of these adverse events were similar to those reported by other studies [10,12,17,18,19,21].

A quite unexpected finding from our survey was significantly better neurological outcome at the 30-day follow-up in patients who were managed with eptifibatide. Theoretically, since this antiplatelet agent was given only to patients with unsuccessful first balloon angioplasty and thus generally with an unfavorable status of the target artery, a worse clinical outcome should be expected. Perhaps, these better clinical outcomes in the eptifibatide group resulted from lower rates of thrombotic and hemorrhagic complications in comparison with patients not receiving this drug (even if these differences in our material were not statistically significant). Theoretically, these differences could be associated with fewer thrombotic occlusions in the cerebral microvasculature [16,22,23,24]. Of note, reocclusion of the ICA or suboptimal thrombectomy are independent predictors of the intracranial bleeding in the settings of ischemic stroke [16,26]. In the study by Renu et al., it was found that the highest rate of bleeding complications occurred in the group in which stent implantation was used in combination with antiplatelet drug, but there was either a suboptimal intracranial recanalization (TICI ≤ 2a) or there was stent thrombosis [23]. Probably, incomplete recanalization and damage to the blood–brain barrier, in combination with the antiplatelet drugs, increased the risk of intracranial bleeding [26].

Considering the retrospective nature of this study, our results should be interpreted with caution; perhaps all stroke patients with tandem lesions should be managed with a low dose of eptifibatide. Indeed, such an approach was reported by Jost et al. They were giving this antiplatelet agent in all tandem lesion patients with stroke irrespective of whether they were treated with stent placement or angioplasty [21]. These authors also recommend the dose of 1 μg/kg/min of eptifibatide, since they have found that the dosing of 0.5 μg/kg/min was associated with high reocclusion rates, while other studies suggested that 2 μg/kg/min can result in an increased risk of potentially fatal intracranial bleeding.

Our study showed that the stenting of extracranial ICA lesions and intracranial thrombectomy augmented with low-dose eptifibatide can be a good strategy for the management of difficult to recanalyze tandem lesions. Perhaps such a medication should be given to all and not only selected tandem lesion patients. A similar beneficial effect of the low dose of eptifibatide in tandem lesion patients has recently been demonstrated in the retrospective analysis by Waters et al. [27]. Still, prospective trials should unequivocally demonstrate safety and efficacy of eptifibatide or other short half-life antiplatelet agents in these challenging stroke patients. Such trials should also establish the optimal dose of this antiplatelet agent. For the time being, no such data are available. Of note, other antiplatelet agents, such as aspirin, clopidogrel, prasugrel or tirofiban, have also been proposed for the management in tandem lesion stroke patients. Still, evidence coming from high-quality clinical trials is scarce, and there is no general consensus regarding the use of these drugs in this particular subgroup of stroke patients [16,28]. We acknowledge that there are important limitations of our survey, comprising its retrospective character, relatively small number of patients assessed and the high heterogeneity of the cohort studied.

## 5. Conclusions

The administration of a modified low dose of eptifibatide in stroke patients presenting with tandem lesions is relatively safe. Moreover, treatment with this drug can possibly improve clinical outcomes in these patients. Yet, prospective randomized clinical trials are needed to unequivocally evaluate the clinical value of this antiplatelet agent.

## Figures and Tables

**Table 1:** Clinical characteristics of the patients, lesion characteristics and endovascular techniques used. NS—difference statistically insignificant.

	Patients Receiving Eptifibatide (*n* = 115)	Patients Not Receiving Eptifibatide (*n* = 33)	*p* Value (Fisher Exact Test)
Age (years), mean (SD)	65.0 (12.7)	63.5 (14.1)	NS
Males/females—*n* (%)	86/29 (75/25)	28/5 (85/15)	NS
Medical history			
hypertension—*n* (%)	67 (58)	18 (55)	NS
diabetes mellitus—*n* (%)	42 (37)	6 (18)	NS
coronary disease—*n* (%)	30 (26)	6 (18)	NS
history of myocardial infarction—*n* (%)	19 (17)	5 (15)	NS
NIHSS at admission—mean (SD)	14 (5.3)	16 (4.7)	NS
ASPECT at admission—mean (SD)	8 (1.5)	7 (1.4)	NS
Extracranial carotid lesion			
Stenosis ≥ 80%—*n* (%)	28 (24)	9 (27)	NS
Occlusion—*n* (%)	87 (76)	24 (73)	NS
Intracranial occlusion location			
Middle cerebral artery or anterior cerebral artery—*n* (%)	78 (68)	25 (76)	NS
Intracranial part of ICA—*n* (%)	37 (32)	8 (24)	NS
Intracranial thrombectomy technique			
stent retriever—*n* (%)	33 (29)	13 (39)	NS
aspiration alone—*n* (%)	82 (71)	20 (61)	NS
Extracranial carotid procedure			
Angioplasty alone	4	33	
Stenting	111	0	

**Table 2:** Complication rates and clinical status at the 30-day follow-up. NS—difference statistically non-significant; * patients who have died during 30-day follow-up were excluded; details regarding statistical test used are given in statistical analysis.

	Patients Receiving Eptifibatide (n = 115)	Patients Not Receiving Eptifibatide (n = 33)	Statistical Significance
Stent and/or ICA thrombosis	6 (5.2%)	5 (15.2%)	NS
Intracranial bleeding	11 (9.5%)	5 (15.2%)	NS
Intracranial bleeding or thrombosis	16 (13.9%)	9 (27.3%)	NS
30-day mortality of any cause	9 (7.8%)	3 (9.1%)	NS
Mortality due to stroke	4 (3.5%)	2 (6.1%)	NS
Median duration of hospital stay duration (days) *	9 (3–38)	10 (4–57)	NS
Median score of the National Institutes of Health Stroke Scale at baseline	15 (2–23)	16 (5–24)	NS
Median score of the National Institutes of Health Stroke Scale at 30-day follow-up *	3 (0–36)	7 (0–21)	NS

**Table 3:** Neurological status of the patients at 30-day follow-up. *p* value calculated with the Mann–Whitney U-test was 0.04.

	Patients Receiving Eptifibatide (n = 115)	Patients Not Receiving Eptifibatide (n = 33)
Patients without neurologic symptoms (NIHSS score: 0)	26 (22.6%)	3 (9.1%)
Patients with mild stroke symptoms (NIHSS score: 1–4)	41 (35.7%)	9 (27.3%)
Patients with moderate stroke symptoms (NIHSS score: 5–14)	31 (27.0%)	15 (45.5%)
Patients with severe stroke symptoms (NIHSS score: 15–24)	7 (6.1%)	3 (9.1%)
Patients with very severe stroke symptoms (NIHSS score: ≥25)	1 (0.9%)	0
Death before 30-day follow-up	9 (7.8%)	3 (9.1%)

## Data Availability

Anonymized data presented in this study are available on request from the corresponding author. Preprint version of this paper is available at https://doi.org/10.20944/preprints202312.2305.v1.
