# Correction: Latacz et al. Safety and Efficacy of Low-Dose Eptifibatide for Tandem Occlusions in Acute Ischemic Stroke. *Neurol. Int.* 2024, *16*, 253–262

**DOI:** 10.3390/neurolint17060090

**Published:** 2025-06-11

**Authors:** Paweł Latacz, Tadeusz Popiela, Paweł Brzegowy, Bartłomiej Lasocha, Krzysztof Kwiecień, Marian Simka

**Affiliations:** 1 Department of Vascular Surgery and Angiology, Brothers of Mercy St. John of God Hospital, 31-061 Krakow, Poland; pawlat@me.com (P.L.); krzyskwiecien@yahoo.com (K.K.); 2 Chair of Radiology, Jagiellonian University Medical College, 31-008 Krakow, Poland; msjpopie@cyf-kr.edu.pl (T.P.); pawelbrzegowy007@gmail.com (P.B.); 3 Diagnostic Imaging Unit, University Hospital, 31-501 Krakow, Poland; blasocha@su.krakow.pl; 4 Institute of Medical Sciences, University of Opole, 45-040 Opole, Poland

## Text Correction

There was an error in the original publication [1]. A correction has been made to the Introduction. Originally, it stated: “Epifibatide is cleared by the kidneys, and its plasma half-life is about 1.5–2 h. Because of its short half-life, in patients without renal insufficiency, a cessation of epifibatide infusion restores normal hemostatic platelet function within 15–30 min [20].”.

This paragraph should be as follows: “Epifibatide is cleared by the kidneys, and its pharmacokinetic plasma half-life is about 4 h. Because of its short half-life, in patients without renal insufficiency, a cessation of epifibatide infusion relatively quickly restores normal hemostatic platelet function [20]. Its biological half-life is estimated to be at the level of 2.5 h, while the “real-life” stop of a prolonged bleeding, demonstrated in many clinical trials and in every-day hospital practice, is within 30 min.”.

The authors state that the scientific conclusions are unaffected. This correction was approved by the Academic Editor. The original publication has also been updated.
